# Research on anterior minimally invasive approach in the treatment of children with developmental dysplasia of the hip

**DOI:** 10.1186/s12891-023-06582-9

**Published:** 2023-06-13

**Authors:** Xiongke Hu, Qian Tan, Haibo Mei, Shasha Mo, Kun Liu

**Affiliations:** 1 Department of Pediatric Orthopedics, Hunan Children’s Hospital, Pediatric Academy of University of South China, No.86, Ziyuan Road, Yuhua District, Changsha, 410000 Hunan Province China; 2 Hunan Provincial Key Laboratory of Pediatric Orthopedics, Changsha, 410008 Hunan China

**Keywords:** Developmental Dysplasia of the hip, Anterior Approach, Open reduction, Hip Joint, Surgical Treatment, IV retrospective study

## Abstract

**Objectives:**

To investigate the clinical efficacy and safety of open reduction through anterior minimally invasive approach in the treatment of children with developmental dysplasia of the hip.

**Method:**

A total of 23 patients (25 hips) less than 2 years with developmental dysplasia of the hip treated by open reduction through anterior minimally invasive approach were treated in our hospital from August 2016 to March 2019. Through the anterior minimally invasive approach, we enter from the gap between sartorius muscle and tensor fasciae lata without cutting off rectus femoris muscle, which can effectively expose the joint capsule and reduce the damage to medial blood vessels and nerves. The operation time, incision length, intraoperative bleeding, hospital stay and surgical complications were observed. The progression of developmental dysplasia of the hip and avascular necrosis of the femoral head were evaluated by imaging examination.

**Result:**

All patients were performed with follow-up visit for an average of 22 months. The average incision length was 2.5 cm, the average operation time was 26 min, the average intraoperative bleeding was 12ml, and the average hospital stay was 4.9 days. All patients received concentric reduction immediately after operation, and no re-dislocation occurred. At the last follow-up visit, the acetabular index was (25.8 ± 6.4°). During the follow-up visit, X-ray showed avascular necrosis of the femoral head in 4 hips (16%).

**Conclusion:**

open reduction through anterior minimally invasive approach can achieve good clinical effect in the treatment of infantile developmental dysplasia of the hip.

Developmental dysplasia of the hip (DDH) is one of the most common musculoskeletal developmental disorders in newborns, complicated with various distortions of hip joint structure, and abnormal acetabulum of femoral head and relaxation of surrounding ligaments [1]. In recent years, with the vigorous promotion of prenatal screening and B-ultrasound technology, the infant DDH incidence rate has gradually increased. The treatment of DDH is mainly to restore the concentric relationship between femoral head and acetabulum, promote the proper development of femoral head and acetabulum, prevent avascular necrosis (AVN) of femoral head and further corrective surgery (FCS). Pavlik sling and Tubingen brace are considered to be effective methods for the treatment of DDH in infants less than 6 months old [2, 3]. At present, it is generally considered that open reduction should be considered for children with DDH over 6 months old who cannot achieve stable reduction through conservative treatment [3, 4]. However, it is still controversial to choose an appropriate surgical method for infant DDH with unstable conservative reduction.

Medial and anterior approaches are the most common open reduction methods for DDH. Open reduction through the medial approach can directly reach the area that hinders the reduction of the hip without invading the abductor muscle of the hip [5]. However, medial approach is easy to damage the medial femoral circumflex artery and obturator nerve, which leads to serious complications such as AVN [6, 7]. Open reduction through anterior approach can shrink the hip joint capsule and remove obstacles in the joint. Although, it will be convenient for pelvic osteotomy, it will come with the risk of local sensory retardation after lateral femoral cutaneous nerve injury [8, 9]. As for the conventional anterolateral approach, it is characterized by large incision required, wide-range exposure, large trauma, unsightly incision and other shortcomings. In order to select a better approach for open reduction, reduce the surgical risk and meet the needs of people for minimally invasive treatment, we designed an anterior minimally invasive approach according to the characteristics of infants. In this study, the operation mode of the anterior minimally invasive approach is described in detail, and the clinical efficacy and the incidence of related complications are analyzed.

## Method

After obtaining the approval of the ethics committee in the hospital, a total of 23 patients (25 hips) with DDH admitted to our hospital from August 2016 to March 2019 were treated with open reduction through anterior small incision, and the collected data were retrospectively studied. Inclusion criteria: ① patients with DDH who were in failure of unsatisfactory reduction or re-dislocation of the child after treatment with a Pavlik harness or closed reduction post-support/plaster fixation; ② patients who treated with open reduction through anterior minimally invasive approach; ③ patients less than 2 years old. Exclusion criteria: ① patients with dislocation of hip joint caused by other reasons such as neuromuscular diseases and trauma; ② patients without follow-up visit.

### General information

A total of 23 patients were included in this study in accordance with the above inclusion criteria, including 14 female patients and 9 male patients (Table 1). The data collected included operation time, incision length, intraoperative bleeding, hospital stay, and complications. The radiographic results were evaluated by measuring acetabular index based on preoperative and postoperative imaging data, as well as IHDI (International Hip Dysplasia Institute) and AVN classification.

**Table 1:** Patient characteristic

Observation index	23 patients (25 hips)
Gender (female/male)	14/9
Age at diagnosis (month)	12 (3–19)
Side (left/right/ bilateral)	9/12/2
Age at surgery (month)	14 (6–20)
Previous treatment (Pavlik harness/Closed reduction)	6/17
Incision length (cm)	2.5 (2.1–3.2)
Operation time (min)	26 (18–45)
Intraoperative blood loss (ml)	12 (5–28)
Follow-up duration (month)	22 (18–40)

### Operation

The surgical position was supine position, and the sheet was disinfected conventionally. After satisfactory anesthesia, the closed reduction of hip joint was tried to be conducted. If the safe zone of Ramsay was less than 30°, open reduction would be required. The patients were injected with 1ml of " iopamidol” in the hip joint through puncture. If the angiography showed that the glenoid labrum of acetabulum and labrum rolled in, with a large number of built-in objects in the hip joint, and the hip joint could not reach the central reduction, open reduction was decided. Similarly, after attempting a closed reduction, we measure the width of the medial dye pool to determine if there is soft tissue trapped between the femoral head and the acetabulum.

**Fig. 1:**
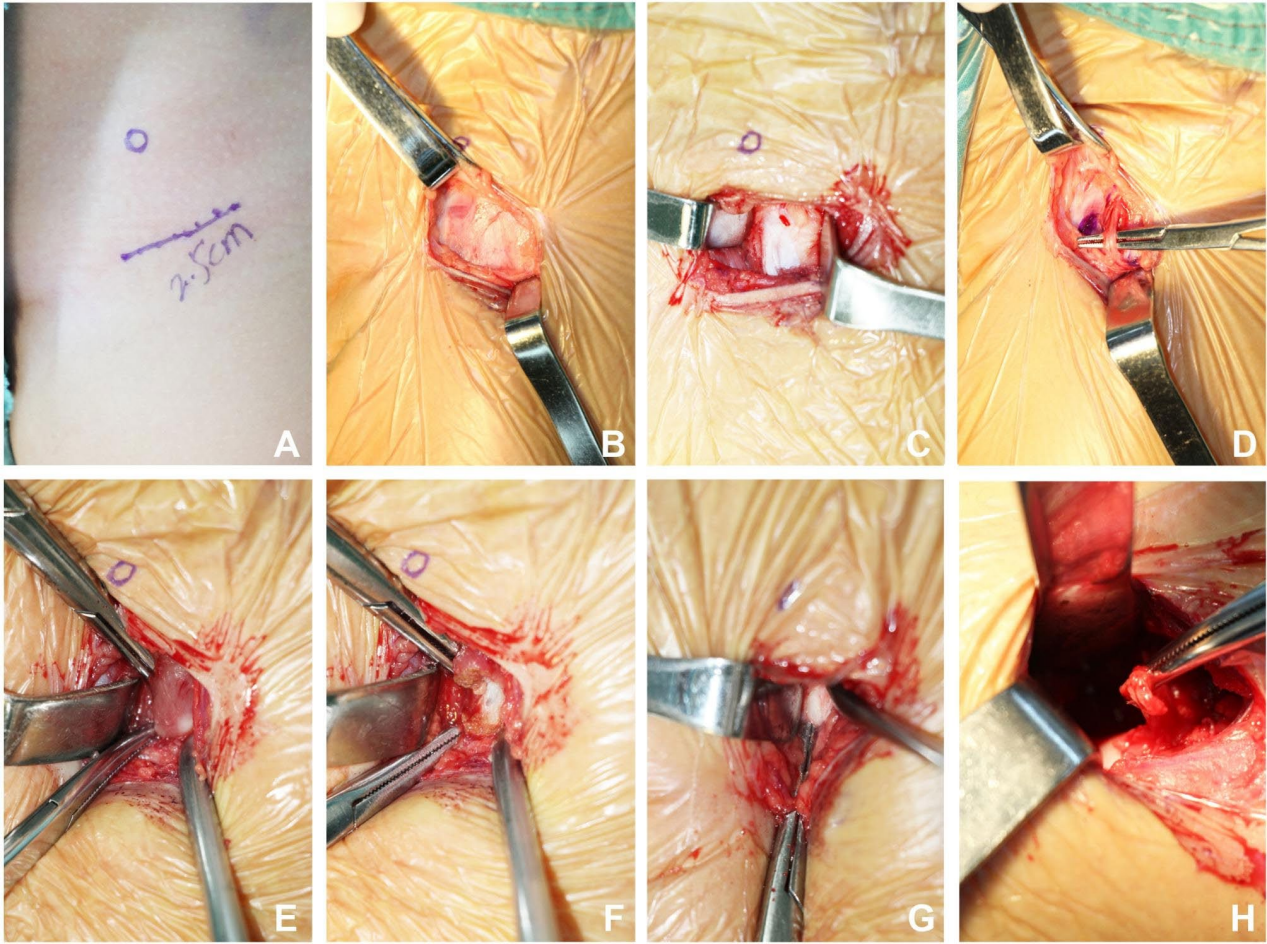
Intraoperative gross photograph **(A)** Incision length is 2.5 cm and the direction of the circle is the anterior superior iliac crest; **(B)** The subcutaneous tissue of the skin was incised; **(C)** Separation was conducted along the gap between sartorius muscle and tensor fasciae lata; **(D)** Separate and protect the lateral femoral cutaneous nerve; **(E)** Expose iliopsoas muscle; **(F)** Disconnect iliopsoas muscle; **(G)** Expose the joint capsule and cut along the acetabular; **(H)** Expose the femoral head and thickened round ligament

An oblique incision with the length of 2-3 cm below the midpoint of the groin was made, the subcutaneous tissue of the skin was incised, and bleeding was stopped with an electrotome (Fig. 1). Separation was conducted along the gap between sartorius muscle and tensor fasciae lata to protect the lateral femoral cutaneous nerve. The rectus femoris muscle was exposed and pulled laterally, and the femoral artery sheath was pulled medially to be protected. The exposed tense iliopsoas tendon was cut off to be released. The anteromedial joint capsule was separated, exposed and cut along the acetabular arc to examine the morphology of the femoral head. The hypertrophic round ligament of the femoral head was incised, the transverse ligament of acetabulum was cut off, and the filling tissue in acetabulum was removed. Subsequently, check whether the femoral head can be easily incorporated into the acetabulum and whether the femoral head can contact the bottom of the acetabulum in the mild internal rotation position of the lower limbs. Then the glenoid lip was incised radially. Check the stability of the hip joint after reduction, its safety range is usually greater than 45°. C-arm fluoroscopy was used to examine the central reduction of the hip joint and the coverage of the femoral head. The incision was rinsed with normal saline, the instruments and dressings were checked and counted. The joint capsule was not sutured, while the deep fascia, subcutaneous tissue and skin were sutured.

After the procedure, the patients kept the flexion of the bilateral hip joint at 95° and abduction at 45° for external fixation with human position hip abduction polymer bandage. After plaster fixation, we observed under C-arm X-ray fluoroscopy whether the central position of the femoral head epiphysis after reduction is located in the inner lower region of the Perkin quadrant, whether the Shenton line is continuous, and whether it meets the concentric reduction of the femoral head and acetabulum. Based on the above observations, if the reduction is not satisfactory. Consider whether there are factors that hinder the reduction, such as incomplete cleaning of the occupying tissue in the acetabulum, incomplete incorporation of the femoral head into the acetabulum, or a gap that is too wide, etc., and make adjustments and perform the repositioning again. The spica cast was fixed for 6–10 weeks, and then the abduction brace was fixed for 12 weeks.

### Statistical analysis

All data were expressed as mean ± standard deviation, and SPSS version 22.0 was used for statistical analysis. Paired t-test was used to evaluate statistical differences, and *P* < 0.05 means that it is statistically significant.

## Result

**Fig. 2:**
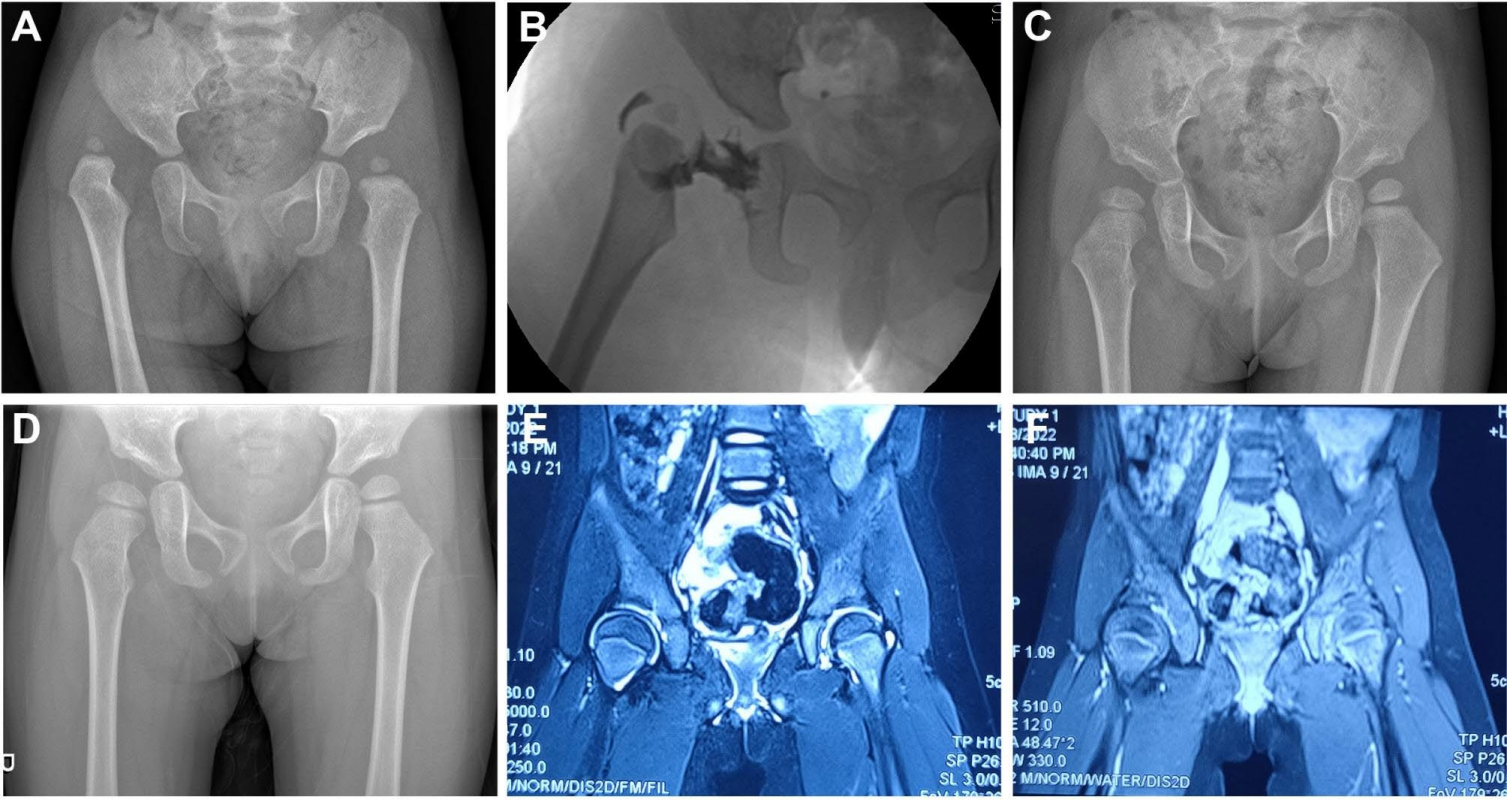
**A.** A 10-month-old female patient was admitted to the hospital due to inconsistency of bilateral breech lines, and was diagnosed as DDH. The preoperative acetabular index was 42.1°, IHDI was type IV; **B.** The pictures of arthrogram during operation; **C.** The follow-up of 14 months after operation showed that the reduction of the right hip joint was well restored, and the acetabular index was 29.2°, IHDI was type I; **D.** 2.5 years after surgery, X-ray showed that the right femoral head developed well, and the acetabulum was slightly dysplasia. **E-F.** 32 months after surgery, MRI showed good hip joint position and no ischemic necrosis of the femoral head

All the patients were conducted with follow-up visit for an average of 22 months. The average incision length was 2.5 cm, the average operation time was 26 min, the average intraoperative bleeding was 12ml, and the average hospital stay was 4.9 days. All the patients received concentric reduction immediately after operation, without re-dislocation. In terms of clinical outcomes, none of the patients had limitation of hip motion, either on the affected or healthy side. All the parents were satisfied or very satisfied with the post-operative appearance. The acetabular index improved from (32.7 ± 2.5°) before operation to (25.5 ± 3.9°) at the last follow-up visit, with a significant difference (*P* < 0.05) (Fig. 2). Preoperative IHDI classification included 7 cases of type III and 18 cases of type IV, while postoperative IHDI classification included 20 cases of type I and 5 cases of type II. At the last follow-up visit, AVN was found in 4 hips (16%), all of which were IHDI type IV (Table 2). All the patients had no complications such as re-dislocation of hip joint and wound infection, and the postoperative hip joint activity was basically normal.

**Table 2:** Radiographic Results

	Preoperative	Postoperative
Acetabular index	32.7 ± 2.5	25.5 ± 3.9^*^
IHDI (I/II/III/IV)	0/0/7/18	20/5/0/0
AVN	0	4

^*^ Indicates a statistically significant difference between preoperative and postoperative of acetabular index (*P* = 0.0037)

## Discussion

DDH is one of the most common musculoskeletal problems in newborns. It is generally believed that open reduction is necessary for patients who have undergone conservative reduction before the age of 2 years. At present, there are two main approaches for open reduction of DDH, including medial approach and anterolateral approach. At present, the commonly used medial approaches include Ludloff anterior medial approach and Ferguson posterior medial approach [10, 11]. As for the medial approach, it usually passes through the medial incision parallel to the adductor muscle to reach the upper part of the joint capsule through the space between the iliopsoas muscle and the pectineal muscle. The advantage of this approach is that the operation field is clear, but it often needs to expose the medial femoral vascular nerve bundle, which is easy to damage the anteromedial femoral circumflex artery of the joint capsule, resulting in AVN. As early as the 1980s, Carl Hueter reported the direct anterior approach to the hip joint, and the approach was then widely promoted and used by Smith Petersen for pelvic and pediatric surgery [12]. Subsequently, a large number of scholars have proposed a variety of new or improved methods of surgeries through anterior approach [13–17]. Both methods can be used to achieve concentric reduction of the femoral head and remove the obstacles to reduction, but the anterior approach can also be combined with pelvic osteotomy if necessary.

As for the classic “Smith-Peterson” approach to the hip joint, it reaches to the anterior superior iliac spine along the middle of the iliac crest firstly, then down across the greater trochanter of the femur, and points to the lateral side of the patella along the lateral side [12]. As for the “Smith-Peterson” approach, it enters through the gap between the sartorius muscle and the tensor fasciae lata muscle, and reaches to the deep rectus femoris and gluteus medius. In order to fully expose the joint capsule, it is necessary to cut off the rectus femoris muscle above, and then separate and pull the iliopsoas muscle below. This surgical approach can be used to well expose the hip joint and iliac bone, and it is applicable to almost all hip surgeries. However, for the surgical approach, it is also traumatic (with incision of about 15 cm for adults and 8-12 cm for children), requires extensive muscle dissection, with poor exposure of the acetabulum. Furthermore, it comes with the risk of damaging the lateral femoral cutaneous nerve and lateral femoral circumflex artery. On this basis, other scholars put forward many improved “Smith-Peterson” approaches [18, 19].

In recent years, with the development of the concept of minimally invasive surgery, we have explored whether infantile DDH can be treated with smaller wounds. In this anterior approach we designed, a minimally invasive incision of about 2-3 cm with the inguinal midpoint inclined downward was selected. Through the gap between sartorius muscle and tensor fasciae lata muscle, the superficial layer of the surgical incision reaches to the rectus femoris muscle to pull apart the rectus femoris muscle from femoral artery sheath. At this time, the children with DDH often had tension of iliopsoas muscle, which hinders the reduction of the femoral head. Therefore, we selected to disconnect the iliopsoas muscle, separate and expose the anteromedial joint capsule, and cut along the acetabular arc. Afterwards, the hypertrophic round ligament of the femoral head was cut off, the transverse ligament of the acetabulum was cut off, and the filling tissue in the acetabulum was removed. After checking the reduction and stability of the femoral head, we conduct the fixation of the hip in the manner of herringbone plaster.

The surgical incision we designed has the average length of 2.5 cm, the direction consistent with the groin dermatoglyphics, almost without problem associated with wound beauty. Our procedure time was 26 min, which is generally consistent with the 22 and 29.8 min reported by other scholars in comparison [17, 20]. Also, in terms of intraoperative bleeding, we were 12 ml, which is close to 9.8 ml and 11.6 ml reported by other scholars [17, 20]. Therefore, we believe that this approach has the advantages of convenient operation, less intraoperative bleeding, and short scars. The importance should be attached to AVN or FCS risk of DDH children after the completion of the reduction. Our anterior minimally invasive approach is actually also part of the modified Smith-Peterson approach, which enters close to the midpoint of the groin, unlike the bikini approach reported by Jia et al. [17]. Also, since our approach does not require the disconnection of the rectus femoris muscle, it allows better maintenance of hip flexion and extension function [21, 22]. We only pull the rectus femoris muscle to one side during the operation, which can reduce damage to the blood vessels in the muscle layer and help protect the blood supply of the femoral head. The incidence of AVN after open reduction of DDH ranges from 4 to 66% [23]. Recent studies have confirmed that preserving the rectus femoris can reduce the incidence of severe AVN after open reduction of DDH [17, 20]. Moreover, we were incised anteromedial to the joint capsule, and the choice of not suturing the joint capsule was beneficial in reducing the incidence of AVN. Our postoperative incidence of AVN is 16%, which is relatively low among all reports of anterior reduction treatment for DDH [20, 23]. These 4 patients with AVN gradually recovered after weight-bearing reduction and conservative treatment, and did not progress to severe femoral head destruction. Additionally, from our experience, the joint capsule does not need to be sutured. The unsutured joint capsule was confirmed to achieve healing at 24 weeks postoperatively [24]. Meanwhile, A randomized controlled trial also confirmed that the closure of the joint capsule is not necessary for open reduction surgery for DDH [25]. The usual direction of dislocation of the femoral head in children was lateral, posterior and superior. We perform an intraoperative medial incision in the anterior aspect of the joint capsule and perform a medial release to remove the obstructing factors and release the pressure. The chance of outward and backward prolapse of the femoral head after repositioning is reduced, and the capsule suture may be chosen not to be performed. Secondly, the patient will need to be fixed with herringbone plaster after operation. After 6 weeks, the plaster will be replaced once. The plaster fixation will last for 3 months, and then the brace will be protected for 6 months. Finally, not suturing the joint capsule is beneficial in reducing the intracapsular pressure and decreasing the chance of AVN. With combination of the protection of plaster and brace, the re-dislocation of femoral head can be effectively avoided. Up to now, all the patients have no re-dislocation of hip joint or poor coverage of femoral head, which need further surgical intervention. However, the study results in the incidence of AVN and FCS were not significantly representative because our follow-up visit was too short. As we all know, the final results of DDH after open reduction can only be evaluated after musculoskeletal growth, therefore, we recommend long-term follow-up visit for all patients [26]. However, our anterior minimally invasive approach also has shortcomings, and it is difficult to expose the medial side of the acetabulum.

We found that open reduction through the anterior minimally invasive approach can achieve good results in the treatment of infant developmental dysplasia of the hip, and it has the advantages of convenient operation, less trauma and fewer complications. However, obvious limitations exist in this study. Firstly, because it is a retrospective study, the collection of preoperative clinical and imaging data is lack of unity, and there is a certain selection bias. Secondly, the follow-up time of this study is too short and the sample size is small. Finally, the evaluation of Radiology indexes and lower limb functional movement of infants is greatly affected by subjective factors. Therefore, a more comprehensive radiology classification system and functional evaluation indexes should be included in our study.

## Conclusions

This study introduced an open reduction through anterior minimally invasive for the treatment of DDH patients and achieve good clinical results. It is a safe approach that can produce short-term results comparable to traditional approaches, with short procedure time, less blood loss and less scarring.

## Data Availability

The datasets used and/or analyzed during the current study are available from the corresponding author on reasonable request.
